# SARS‐CoV‐2 mRNA Vaccination Induces Neutralizing Antibodies and Type I IFN Changes in People Living With HIV

**DOI:** 10.1002/jmv.71067

**Published:** 2026-07-27

**Authors:** Federica Frasca, Luca Maddaloni, Alessandra D'Auria, Matteo Fracella, Ginevra Bugani, Carmen Falvino, Valentina Baccolini, Giuseppe Migliara, Francesca Aloisi, Letizia Santinelli, Enrico Palermo, Luca Bortolani, Alessandro Lazzaro, Giancarlo Ceccarelli, Ombretta Turriziani, Claudio Maria Mastroianni, Guido Antonelli, Gabriella d'Ettorre, Carolina Scagnolari

**Affiliations:** ^1^ Department of Health and Life Sciences European University of Rome Rome Italy; ^2^ Laboratory of Virology, Department of Molecular Medicine Sapienza University of Rome Rome Italy; ^3^ Department of Public Health and Infectious Diseases Sapienza University of Rome Rome Italy; ^4^ Laboratory of Virology National Institute for Infectious Diseases “Lazzaro Spallanzani” IRCCS Rome Italy; ^5^ Department of Life Sciences, Health and Health Professions Link Campus University Rome Italy; ^6^ Istituto Pasteur Italia‐Cenci Bolognetti Foundation Rome Italy; ^7^ Microbiology and Virology Unit, Policlinico Umberto I Hospital Sapienza University of Rome Rome Italy

**Keywords:** anti‐Spike antibodies, BNT162b2 COVID‐19 vaccine, HIV, interferon

## Abstract

This study examined changes in anti‐Spike (anti‐S) antibodies (Abs) and type I interferon (IFN‐I) following the BNT162b2 vaccine in people living with HIV (PLWH) and analyzed the impact of demographic and immunological factors. In total, 75 PLWH and 28 healthy donors were followed at baseline (T0), at the second dose (T1), after the second dose (T2), and more than 1 year later (T3). Anti‐S Abs were assessed by chemiluminescence and vesicular stomatitis virus (VSV)‐based pseudo virus‐neutralization assay, while IFN‐α2, IFN‐β, and IFN‐ω mRNA levels were measured by RT‐Real Time PCR. PLWH showed an increase in anti‐S Immunoglobulin G (IgG) levels comparable to healthy donors (*p* < 0.001) and an induction of anti‐S neutralizing Abs (*p* < 0.014 for T2 vs. T3). Age, gender, CD4^+^ T cell count, exposure to combined antiretroviral therapy (cART) and IFN‐I levels at T0 did not affect the anti‐S IgG production. IFN‐I gene expression showed temporal changes, with a decrease at T2 (*p* < 0.01) and a subsequent increase at T3 (*p* < 0.001, for IFN‐α2 and IFN‐ω). A multivariable model revealed no overall change in the IFN‐I response over time, except for IFN‐β, which was lower at T3 than at T1 (*p* = 0.032). CD4^+^ T cell count was positively correlated with the IFN‐I response (*p* < 0.05). These results suggest that mRNA vaccination can elicit an effective anti‐S response and modulate the IFN‐β gene expression in PLWH, with CD4^+^ T cell count being a key determinant of vaccine‐induced changes in IFN.

## 1 Introduction

Vaccines have proven to be the most effective control measure for severe acute respiratory syndrome coronavirus 2 (SARS‐CoV‐2), reducing morbidity and mortality from the resulting disease, coronavirus disease 2019 (COVID‐19) [1]. Anti‐SARS‐CoV‐2 vaccines stimulate a strong adaptive immune response against the spike (S) glycoprotein of SARS‐CoV‐2, producing antibodies and activating T cells. This enables the body to maintain immunological memory against SARS‐CoV‐2 [2, 3]. Vaccination is particularly important for frail patients and individuals at risk of developing severe disease, such as people living with HIV (PLWH). Although the SARS‐CoV‐2 positivity rate in PLWH is similar to that observed in HIV‐negative individuals, PLWH may have a worse prognosis, particularly if they have a low CD4^+^ T cell count or comorbidities [4, 5]. Some aspects of the efficacy of vaccination in PLWH have been evaluated, and the few existing studies seem to indicate a good humoral and cell‐mediated response. However, this appears to be highly influenced by immune status [6, 7].

Recent evidence has revealed a link between vaccines and innate immunity, allowing the latter to no longer be considered merely a “first line of defense,” but rather as having the capacity to actively influence the quality of the antibody response [8, 9]. Interferons (IFNs) are a critical component of innate immunity, playing a key role in the initial defense against viral infections by orchestrating a complex antiviral response. They are also important in stimulating the adaptive immune response [10]. Regarding the relationship between IFN and vaccination response, inactivated nonadjuvanted trivalent influenza vaccine (TIV) has been shown to trigger an early IFN response in dendritic cells (DCs), which correlates with antibody response [11]. Otherwise, the important role of IFN in regulating innate immunity and influencing the magnitude of the adaptive response after SARS‐CoV‐2 vaccination has recently been highlighted. Indeed, the vaccine‐induced early regulation of innate immunity is mediated by the IFN signaling and affects the magnitude of the humoral response [12]. Moreover, the CD8^+^ T cell response induced by the mRNA‐based SARS‐CoV‐2 vaccine was found to depend on melanoma differentiation‐associated protein 5 (MDA5) signaling, which is mediated by type I IFN (IFN‐I) [13]. However, IFN plays a dichotomous role in the context of HIV infection. While it inhibits HIV replication in the acute phase, its sustained expression in the chronic phase plays a key role in pathogenesis by inducing persistent immune system activation [14, 15].

The potential of these vaccines to modulate innate immunity in PLWH remains largely unexplored. Therefore, in order to improve our understanding of the immunogenicity of SARS‐CoV‐2 vaccines in PLWH, this study aimed to investigate, for the first time, whether vaccination induces anti‐Spike (anti‐S) antibody production and changes IFN‐I expression in this population. Given the role of IFN‐I in stimulating B and T lymphocyte responses [16, 17], and the recent evidence on the relationship between vaccination, changes in IFN production and adaptive immune responses [18], we also evaluated whether IFN‐I levels could affect anti‐S antibody production in PLWH, as well as host parameters that impact the IFN response during vaccination.

## 2 Materials and Methods

### 2.1 Study Participants

**Figure 1:**
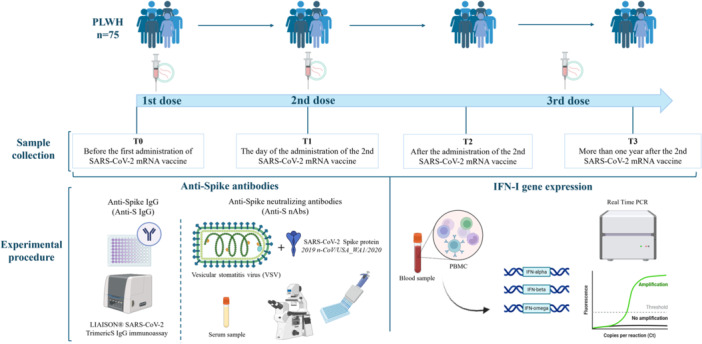
Pipeline of the study. PLWH, people living with HIV; PBMCs, peripheral blood mononuclear cells; IFN, interferon.

In total, 75 PLWH, who were on long‐term combined antiretroviral therapy (cART) and had received three doses of the BNT162b2 (Pfizer‐BioNTech, Comirnaty) COVID‐19 vaccine, were recruited at the Department of Public Health and Infectious Diseases at Sapienza University of Rome, Italy. The inclusion criteria were confirmed HIV‐1 infection, an age of at least 18 years, and vaccination with BNT162b2 vaccine. The exclusion criteria were detectable HIV‐1 RNA in the previous 6 months, prior vaccination against SARS‐CoV‐2, and ongoing pregnancy. Peripheral blood samples (*n* = 300) were collected prospectively at four time points: before the first dose (T0), on the day of the second dose [T1, median (min–max) 21 (13–77) days from T0], after the second dose [T2, 38 (13–204) days from T1], and a late follow‐up time point more than 1 year after T2 [T3, 405 (231–513) days from T2]. In our study group, 73 out of 75 PLWH (97.33%) received a booster, and among them, 16 (21.19%) reported SARS‐CoV‐2 infection after the second dose (Figure 1). Data on positive SARS‐CoV‐2 nasopharyngeal swabs after T2 were obtained from the remaining 2 participants who did not receive a booster vaccine dose. All PLWH enrolled in the study were SARS‐CoV‐2 naïve at baseline, and their infection status was monitored throughout the study. A control group of 28 healthy donors, who were vaccinated with BNT162b2 during the same period, was also included. The study protocol was approved by the institutional Review Board (Department of Public Health and Infectious Diseases, Sapienza University of Rome) and the Ethics Commitee (Comitato Etico Territoriale Lazio Area 1) (Ref. 6260, Prot. 0099/2021). Written informed consent was obtained from each study participant.

### 2.2 Bioassay for Detection of Neutralizing Antibodies Against IFN‐α2, ‐β, and ‐ω

Serum samples were collected and spun at 1500 g for 10 min to remove platelets before freezing at −80°C for later analysis of anti‐IFNs neutralizing antibodies (nAbs). All serum samples were assayed for nAbs to IFN‐α2 subtype (Intron; Schering‐Plough, Kenilworth, New Jersey, USA), IFN‐β (Rebif, Serono, Geneva, Switzerland), and IFN‐ω (PBL Interferon Source, Piscataway, USA) in a bioassay based on the IFN‐induced inhibition of the encephalomyocarditis virus (EMCV) cytopathic effect on human lung carcinoma epithelial cells (A549), as previously reported [19].

### 2.3 Quantification of Anti‐Spike Antibodies Using SARS‐CoV‐2 TrimericS IgG Immunoassay

The antibody response to the BNT162b2 vaccine was assessed using the chemiluminescence technology of LIAISON SARS‐CoV‐2 TrimericS IgG immunoassay (DiaSorin S.p.A.), which allows the quantitative determination of specific IgG antibodies against the SARS‐CoV‐2 Trimeric spike protein in serum samples (Figure 1). The clinical laboratory IgG titers were expressed in binding antibody units/mL (BAU/mL). The detection limit for antibody titers in serum was 4.81 BAU/mL. According to the manufacturer's instructions, values in the range of 4.81–33.8 BAU/mL were considered negative, and values greater than or equal to 33.8 BAU/mL were considered positive. The LIAISON SARS‐CoV‐2 TrimericS IgG immunoassay provided quantitative results, demonstrating 100% sensitivity (95% CI: 85.1–100.0) and 99.5% specificity (95% CI: 99.0–99.7). In Section 3, SARS‐CoV‐2 TrimericS IgG is referred to as “anti‐S IgG.”

### 2.4 Quantification of Anti‐Spike Neutralizing Antibodies Using VSV‐Based Pseudo‐Neutralization Assay

An in vitro virus neutralization assay was performed to assess the presence of anti‐S nAbs in serum samples of PLWH who had received the BNT162b2 vaccine (Figure 1). The assay involves detecting antibodies that can neutralize on Vero E6 the replication‐competent VSV expressing eGFP in the first position of the genome as well as a modified version of the SARS‐CoV‐2 spike (derived from the 2019‐nCoV/WHU01 strain) in place of the native VSV glycoprotein (VSV‐Spike). VSV‐Spike was propagated in Vero E6 cells and harvested after the appearance of cytopathic effects at 72 h post‐infection (hpi) [20]. Virus titer was determined using the Reed and Muench method [21] and expressed as 50% tissue culture infective dose (TCID_50_/mL). The VSV‐Spike pseudo‐neutralization test was performed as previously reported in detail in Wondeu et al. [22]. Three replicates of serum samples were evaluated in serial dilutions ranging from 1:10 to 1:20,480 and neutralization positivity was defined as detectable neutralizing activity at the lowest tested dilution (1:10). In Section 3, anti‐Spike nAbs are referred to as “anti‐S nAbs.”

### 2.5 TaqMan‐Based Real‐Time RT‐PCR Assay for IFN‐Related Gene Expression

Peripheral blood mononuclear cells (PBMCs) were isolated from fresh blood by Lympholyte Cell Separation Media (Cedarlane, Burlington, Ontario, CAN) and collected as dry pellet at −80°C to perform gene expression analysis. Total RNA extracted from PBMCs using the Zymo Research (Irvine, CA, USA) was reverse transcribed using High‐Capacity cDNA Reverse Transcription Kit (Applied Biosystems, Foster City, California, USA), following the manufacturer's protocol. The mRNA levels of IFN‐α2, IFN‐β, and IFN‐ω were analyzed in three replicates for samples with the LightCycler 480 instrument (Roche, Basel, Switzerland) (Figure 1). The mRNA copy numbers of the target genes were measured by co‐amplifying them with the β‐glucuronidase (GUS) gene in order to normalize the amount of total RNA using the threshold cycle (Ct) relative quantification method (2^−ΔCt^ method). Primers and probes for GUS, IFN‐α2, IFN‐β, and IFN‐ω were used as previously reported [23]. For mRNA copies that were undetectable by the RT‐PCR assay, the Ct was set to 45, for calculating the fold changes to the GUS Ct of the same sample. A healthy donor control group (*n* = 46) was included to compare baseline IFN‐I expression levels with those observed in PLWH before SARS‐CoV‐2 vaccination. IFN‐I levels in healthy donors were also assessed prior SARS‐CoV‐2 vaccination. Quality control procedures were applied during analysis, including evaluation of replicate consistency and inclusion of no‐template controls.

### 2.6 Statistical Analysis

Descriptive statistics were obtained using mean (standard deviation, SD) or median [range (min–max)] for continuous variables and proportions (%) for dichotomous and categorical variables. The levels of anti‐S IgG, anti‐S nAbs, and IFN‐α2, IFN‐β, and IFN‐ω were natural log–transformed (ln) prior to analysis to improve distributional properties. The Mann–Whitney *U* test was used to compare continuous variables between male and female PLWH. At each time‐point (T1–T3), the association between anti‐S IgG and anti‐S nAbs, as well as between anti‐S IgG or anti‐S nAbs and IFN‐I gene expression, was assessed using Spearman's rank correlation, reporting the correlation coefficient (*r*) and two‐sided *p* value.

Longitudinal changes in anti‐S IgG, anti‐S nAbs, and IFN‐α2, IFN‐β, and IFN‐ω (all analyzed on the ln scale) were evaluated using population‐averaged generalized estimating equation (GEE) models with a Gaussian family and identity link, specifying an exchangeable working correlation structure to account for within‐participant correlation across repeated measurements and using robust standard errors clustered by participant ID. Time was modeled as a categorical predictor (with the earliest time‐point as reference), and pairwise comparisons between time‐points were obtained from marginal linear predictions with Bonferroni adjustment for multiple testing.

Then, four multivariable population‐averaged GEE models were fitted to investigate independent associations with longitudinal changes in anti‐S IgG levels and IFN gene expression (IFN‐α2, IFN‐β, and IFN‐ω). The following variables were included in the multivariable models, based on expert knowledge: time (i.e., T1, T2, and T3, categorical), age (years, continuous), gender (binary), CD4^+^ T cell count at T0 (continuous), nadir CD4^+^ T cell count (continuous), duration of cART (years, continuous), and days elapsed between time‐points (continuous). Baseline values at T0 were additionally included to control for initial levels (baseline ln anti‐S IgG and baseline ln IFN‐α, ln IFN‐β, and ln IFN‐ω, respectively, in the four models). Model results are reported as β coefficients with 95% confidence intervals (CI) and two‐sided *p* values. A *p* < 0.05 was considered statistically significant. Data collected were analyzed using SPSS statistical software version 20 (IBM, USA) and STATA version 19.0 (StataCorp LLC, 4905 Lakeway Drive, College Station, TX, USA). Graphs were made using GraphPad PRISM 8.0.1 software.

## 3 Results

### 3.1 Characteristics of PLWH Receiving BNT162b2 COVID‐19 Vaccination

Table 1 summarizes the demographic and clinical characteristics of the 75 PLWH who were enrolled in the study. Of these, 50 were males (66.66%), with a mean age of 62 years (±8.93). No significant differences were found when comparing males and females (Table 1). All participants were on long‐term effective cART at the time they received the first BNT162b2 vaccine dose, with an HIV‐1 RNA level lower than 37 copies/mL and had been diagnosed with HIV‐1 for a median of 20 years (min–max: 4–36). Their CD4^+^ T cell count ranged from 188 to 1584 cells/mm^3^. Additionally, 34 out of 75 PLWH (45.33%) reported a history of AIDS‐defining events (Table 1). To exclude confounding factors in the analysis of IFN expression, all serum samples were tested for the presence of anti‐IFN‐I nAbs. All PLWH tested negative for anti‐IFN‐α2/β/ω nAbs (< 10 TRU/mL) at all time‐points.

**Table 1:** Demographic, immunological, and clinical characteristics of cART treated PLWH.

Item	PLWH (*n* = 75)	Males PLWH (*n* = 50) (A)	Females PLWH (*n* = 25) (B)	*p* a A vs. B
Age (years) mean, SD	61 (±8.40)	62 (±8.93)	60 (±7.34)	0.5003
Gender (males/females)	50/25	NA	NA	NA
CD4^+^ T cells/mm^3^ median (min–max)	633 (188–1584)	630 (188–1337)	655 (232–1584)	0.4798
Ratio CD4/CD8 median (min–max)	0.71 (0.22–2.93)	0.69 (0.22–2.31)	0.98 (0.30–2.93)	0.1608
Nadir CD4^+^ T cell count median (min–max)	123 (2–866)	123 (2–866)	115 (22–554)	0.9950
HIV‐1 RNA (copies/mL)^b^	< 37	< 37	< 37	NA
Duration of cART (years) median (min–max)	20 (4–36)	17 (4–36)	21 (9–32)	0.2179
Anti‐HIV‐1 drug class (*N*)	PI (13)	PI (8)	PI (6)	NA
	NRTI (65)	NRTI (44)	NRTI (24)	
	NNRTI (16)	NNRTI (12)	NNRTI (4)	
	INSTI (24)	INSTI (12)	INSTI (13)	
	TAF (53)	TAF (34)	TAF (20)	
(*N*)^c^	Neurotoxoplasmosis (3)	Neurotoxoplasmosis (1)	Neurotoxoplasmosis (2)	NA
	Tuberculosis (5)	Tuberculosis (3)	Tuberculosis (2)	
	Non‐Hodkings lymphoma (6)	Non‐Hodkings lymphoma (4)	Non‐Hodkings lymphoma (2)	
	Pneumocystis jirovecii pneumonia (10)	Pneumocystis jirovecii pneumonia (8)	Pneumocystis jirovecii pneumonia (2)	
	Kaposi's sarcoma (2)	Kaposi's sarcoma (2)	Kaposi's sarcoma (0)	
	Visceral Leishmaniasis (1)	Visceral Leishmaniasis (1)	Visceral Leishmaniasis (0)	
	Oropharyngeal candidiasis (2)	Oropharyngeal candidiasis (2)	Oropharyngeal candidiasis (0)	
	Leukoencephalopathy (1)	Leukoencephalopathy (0)	Leukoencephalopathy (1)	
	Cytomegalovirus (CMV) chorioretinitis (2)	Cytomegalovirus (CMV) chorioretinitis (2)	Cytomegalovirus (CMV) chorioretinitis (0)	
	Luetic uveitis (1)	Luetic uveitis (0)	Luetic uveitis (1)	
	Wasting syndrome (1)	Wasting syndrome (1)	Wasting syndrome (0)	

*Note:* Data are expressed as total number (*N*), mean and standard deviation (SD), percentage (%), and median (min–max). Medical history (i.e., years since cART initiation, administered cART, and AIDS‐defining events) and immune‐virological parameters (i.e., CD4^+^ T cell count at T0, nadir CD4^+^ T cell count, and blood HIV RNA at enrollment) of each participant were collected before starting mRNA‐based SARS‐CoV‐2 vaccination. People living with HIV (PLWH) received three doses of the Pfizer mRNA BNT162b2 (Comirnaty) COVID‐19 vaccine. PLWH were negative to anti‐IFN‐I nAbs. PI drugs: Darunavir, Cobicistat; NRTI drugs: Emtricitabine, Lamuvidine, Abacavir, Tenofovir Disoproxil Fumarate; NNRTI drugs: Rilpivirine, Doravirine; INSTI: Elvitegravir, Bictegravir, Dolutegravir.

Abbreviations: cART, combined antiretroviral therapy; PI, protease inhibitors; NRTI, nucleoside reverse transcriptase T‐inhibitor; NNRTI, non‐nucleoside reverse transcriptase inhibitor; INSTI, integrase strand transfer inhibitor; TAF, tenofovir alafenamide; NA, not available.

a Differences between HIV‐1‐infected patients' categories were evaluated using Mann‐Whitney tests.

b HIV viral load was determined by Versant HIV‐1 RNA kPCR Assay (Siemens Healthcare Diagnostic, Tarrytown NY, USA), which has a limit of detection of 37 copies/mL.

c History of AIDS‐defining events was reported for 34 out of 75 PLWH.

### 3.2 Longitudinal Profile of Anti‐Spike IgG Titers in Serum Samples From PLWH and Healthy Donors

**Figure 2:**
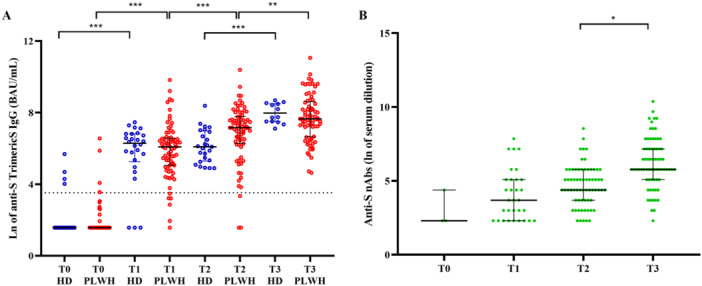
Anti‐SARS‐CoV‐2 humoral immunity in PLWH and healthy donors receiving BNT162b2 COVID‐19 vaccination. (A) The anti‐S trimericS IgG (BAU/mL) measured in the serum samples of people living with HIV (PLWH) and healthy donors (HD) before the first BNT162b2 vaccine administration (T0: PLWH, *n* = 75; HD, *n* = 26), on the day of the second vaccine dose administration (T1: PLWH, *n* = 75, HD, *n* = 26), after the second BNT162b2 vaccine injection (T2: PLWH *n* = 75, HD, *n* = 25), and more than 1 year after T2 (T3: PLWH, *n* = 75, HD, *n* = 14). (B) The mean of three replicates of anti‐S neutralizing antibodies (nAbs) (serum dilution) measured in PLWH. Data of anti‐S TrimericS IgG and anti‐S nAbs are reported as natural logarithm (ln). Anti‐S nAb titers, with median and interquartile range, were considered the highest dilution of plasma samples without evidence of CPE in Vero E6 cells. ∗*p* < 0.05; ∗∗*p* < 0.001; ∗∗∗*p* < 0.0001.

To explore the kinetics of the antibody response in PLWH following BNT162b2 vaccination, we analyzed levels of anti‐S IgG at T0, T1, T2, and T3 (Figure 2A and Table 2). Anti‐S IgG titers increased in all serum samples from PLWH over the study period (T0 vs. T1, T1 vs. T2, and T2 vs. T3: *p* < 0.001) (Figure 2A). Specifically, 6.66% (*n* = 5/75) of serum samples from PLWH had detectable anti‐S IgG levels (IgG ≥ 33.8 BAU/mL) prior to BNT162b2 vaccination initiation (T0), despite none of the patients having reported a history of natural SARS‐CoV‐2 infection (Table 2). Subsequently, 92% (*n* = 69/75) of serum samples from PLWH had detectable anti‐S IgG levels (IgG ≥ 33.8 BAU/mL) at T1, with the positivity rate increasing to 96% (*n* = 72/75) at T2 (Table 2). All sera from PLWH (*n* = 75) showed anti‐S IgG at T3 (Table 2). A similar trend in anti‐S IgG serum levels over time was observed in a group of healthy donors (mean age: 44 years, SD: ±14.31 years; males/females: 5/23; T0 vs. T1 and T2 vs. T3: *p* < 0.0001; Figure 2A). No significant differences in anti‐S IgG levels were observed between serum samples from PLWH and healthy donors at any of the time‐points analyzed (*p* > 0.05) (Figure 2A).

**Table 2:** Levels and frequencies of anti‐Spike IgG and anti‐Spike neutralizing antibodies in serum samples from people living with HIV.

Time‐points analyzed in the study	Anti‐S IgG	Anti‐S nAbs
T0		
[*N*, (%)]	5/75 (6.66)	2/5 (40)
Median (min–max)	59 (35–699)	45 (10–80)
T1		
[*N*, (%)]	69/75 (92)	31/69 (44.92)
Median (min–max)	494 (44–18 500)	40 (10–2560)
T2		
[*N*, (%)]	72/75 (96)	65/72 (90.27)
Median (min–max)	1349 (46–32 600)	80 (10– 5120)
T3		
[*N*, (%)]	75/75 (100)	71/75 (94.66)
Median (min–max)	2100 (103–63 800)	320 (10–3200)

*Note:* The data are expressed as the total number (*N*), percentage (%), and the median titers (min–max). Anti‐Spike IgG (Anti‐S IgG) (BAU/mL) and anti‐Spike neutralizing antibodies (anti‐S nAbs) (serum dilution) have been determined by using the LIASON SARS‐CoV‐2 TrimericS IgG immunoassay and in vitro VSV‐based pseudo‐neutralization test, respectively.

### 3.3 Longitudinal Profile of Anti‐Spike Neutralizing Antibodies in Serum Samples From PLWH

Further testing was conducted on the serum samples of PLWH to confirm the results obtained from the serological assay for detecting SARS‐CoV‐2 IgG, using an in vitro VSV‐Spike‐based pseudo‐neutralization test to detect the presence of anti‐S nAbs (Table 2 and Figure 2B). A trend toward an increase in anti‐S nAb levels was observed in serum samples from PLWH between T1 and T2. Furthermore, anti‐S nAb levels were higher at T3 than at T2 (*p* = 0.014) (Figure 2B). A positive correlation was also observed between anti‐S nAbs and anti‐S IgG at each time‐point analyzed (T1: *p* = 0.0020; *r* = 0.5325; T2: *p* < 0.0001; *r* = 0.7958; T3: *p* < 0.0001; *r* = 0.4892) (Supporting Information S1: Figure 1). Of the five serum samples from PLWH with detectable levels of anti‐S IgG at T0, two (40%) were confirmed by the VSV‐Spike‐based pseudo‐neutralization test (Table 2). Analysis of subsequent time points showed that 44.92% (31/69), 90.27% (65/72), and 94.66% (71/75) of serum samples from PLWH with anti‐S IgG tested positive for anti‐S nAbs at T1, T2, and T3, respectively (Table 2).

### 3.4 Multivariable Analysis of the Impact of Demographic, Medical History, and Immunological Parameters on Anti‐Spike IgG in PLWH

A multivariable analysis was performed in PLWH to assess factors that might influence anti‐S IgG levels during the study period. The analysis adjusted for demographic characteristics (age and gender), time of exposure to cART (years), immunological parameters (nadir CD4^+^ T cell count and CD4^+^ T cell count at T0), baseline anti‐S IgG at T0, baseline IFN levels at T0 (IFN‐α2, IFN‐β, and IFN‐ω) and time interval between measurements (days), confirms a progressive increase of anti‐S IgG over time (T2 vs. T1: *β* = 1.693, 95% CI: 0.900–2.486, *p* < 0.001; T3 vs. T1: *β* = 1.840, 95% CI: 1.350–2.330, *p* < 0.001) (Table 3). Baseline IFN measures at T0 [IFN‐α2 (*β* = 0.144, *p* = 0.515), IFN‐β (*β* = −0.216, *p* = 0.398), and IFN‐ω (*β* = 0.105, *p* = 0.251)] as well as baseline anti‐S IgG at T0 (*β* = 0.019, *p* = 0.894) were not significantly associated with anti‐S IgG over follow‐up (Table 3). Likewise, age (*β* = −0.016, *p* = 0.257), gender (*β* = 0.334, *p* = 0.205), CD4^+^ T cell count at T0 (*β* = −0.001, *p* = 0.497), nadir CD4^+^ T cell count (*β* = −0.001, *p* = 0.973), and exposure to cART (*β* = −0.005, *p* = 0.755) showed no significant associations with outcome (Table 3). However, the time interval between measurements (in days) was the only factor independently associated with lower anti‐S IgG values (*β* = −0.009; 95% CI: −0.015 to −0.003; *p* = 0.004). Additionally, the history of SARS‐CoV‐2 infection after the second dose affects the development of anti‐S IgG (Supporting Information S2: Table 1). However, multivariable analysis with interactions between time points and history of SARS‐CoV‐2 infection after the second dose showed no effects of SARS‐CoV‐2 infection on anti‐S IgG at T3 (Supporting Information S2: Table 2)

**Table 3:** Multivariable analysis of the factors influencing anti‐S IgG production in PLWH.

Variable	*β* coefficient	95% CI	*p*
T2 vs. T1	1.693	0.900 to 2.486	< 0.001
T3 vs. T1	1.840	1.350 to 2.330	< 0.001
IFN‐α at T0 (ln‐transformed)	0.144	−0.289 to 0.576	0.515
IFN‐β at T0 (ln‐transformed)	−0.216	−0.717 to 0.285	0.398
IFN‐ω at T0 (ln‐transformed)	0.105	−0.074 to 0.283	0.251
Age (years)	−0.016	−0.044 to 0.012	0.257
Gender	0.334	−0.182 to 0.850	0.205
Nadir CD4^+^ T cell count (cells/µL)	−0.001	−0.002 to 0.002	0.973
CD4^+^ T cell count at T0 (cells/µL)	−0.001	−0.001 to 0.001	0.497
Exposure to cART (years)	−0.005	−0.037 to 0.027	0.755
Days between measurements	−0.009	−0.015 to −0.003	0.004
Anti‐S IgG at T0 (ln‐transformed)	0.019	−0.264 to 0.302	0.894

Abbreviations: CI, confidence interval; Ln, natural logarithm.

To characterize a direct relationship between IFN‐I gene expression and the SARS‐CoV‐2 mRNA vaccine‐induced humoral responses, we further analyzed a possible correlation between IFN‐I gene expression levels and binding antibody levels or nAb titers at corresponding time points. However, no association between IFN‐α2, IFN‐β, and IFN‐ω and anti‐S IgG as well as anti‐S nAbs was observed at each time point analyzed (Supporting Information S2: Tables 6 and 7).

### 3.5 Longitudinal Evaluation of IFN‐I Gene Expression in PBMCs of PLWH

Given the dual role of the IFN response, together with the sustained IFN dysregulation described in PLWH [24], and recent evidence showing increased expression of IFN‐JAK‐STAT‐regulated antiviral genes after SARS‐CoV‐2 mRNA vaccination in healthy donors [25], we investigated the effect of BNT162b2 vaccination on IFN‐α2, IFN‐β, and IFN‐ω transcript levels in PBMCs from PLWH over the study period. In an initial analysis, baseline IFN‐I gene expression (T0) in PLWH was compared with that in a group of healthy donors. IFN‐α2, IFN‐β, and IFN‐ω expression levels were significantly higher in PLWH than in healthy donors (*p* < 0.001; Supporting Information S1: Figure 2).

**Figure 3:**
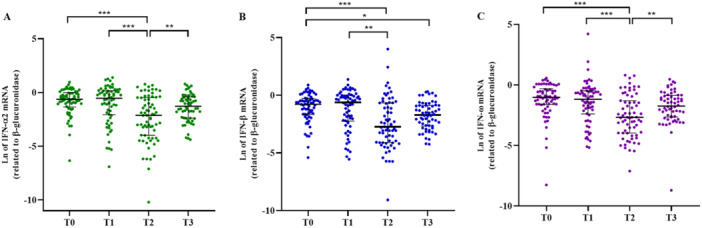
Expression levels of IFN‐I in PLWH receiving BNT162b2 COVID‐19 vaccination. Expression levels of genes encoding IFN‐α2 (A), IFN‐β (B), IFN‐ω (C) measured by RT‐Real Time PCR, in PBMCs collected from people living with HIV (PLWH) before the first administration of BNT162b2 vaccine (T0, *n* = 66), the day of the administration of the second vaccine dose (T1, *n* = 66), after the administration of the second injection of BNT162b2 vaccine (T2, *N* = 67) and then more than 1 year after the T2 time‐point (T3, *n* = 60). The analysis of gene expression differences for IFN‐α2, IFN‐β, and IFN‐ω related to GUS (2^−ΔCt^ method) was conducted using the maximum number of available observations. Data are shown as natural logarithm (ln) and both median values and interquartile range of gene expression levels are reported. ∗*p* < 0.05; ∗∗*p* < 0.001; ∗∗∗*p* < 0.0001.

Regarding the longitudinal evaluation of IFN response in PLWH, no significant differences were detected in the expression of the IFN‐α2, IFN‐β, and IFN‐ω genes between T1 and T0 (Figure 3A–C). By contrast, pairwise comparisons revealed decreased levels of all IFN‐I subtypes at T2 compared to T1 and T0 (*p* < 0.001 for all the genes) (Figure 3A–C). At T3, an induction of IFN‐α2 (Figure 3A) and IFN‐ω (Figure 3C), as well as a trend toward increased IFN‐β expression (Figure 3B), was observed compared to T2 (*p* < 0.001 for IFN‐α2 and IFN‐ω; *p* > 0.05 for IFN‐β). However, IFN‐β mRNA transcript levels measured at T3 were significantly lower than baseline levels at T0 (*p* = 0.020) (Figure 3B). Conversely, the median expression levels of IFN‐α2 (Figure 3A) and IFN‐ω (Figure 3C) at T3 did not differ significantly from those observed at T0 (*p* > 0.05).

### 3.6 Multivariable Analysis to Assess the Impact of Demographic, Medical History, and Immunological Parameters of PLWH on IFN‐I Gene Expression

Multivariable testing was performed to evaluate the impact of independent factors such as demographic data (age and sex), time of exposure to cART (years), immunological parameters (nadir CD4^+^ T cell count and CD4^+^ T cell count at T0), as well as the number of days between each time point on IFN‐I (IFN‐α2, IFN‐β, IFN‐ω) gene expression over the study period. As for the predictors of ln‐transformed IFN‐α2, IFN‐β, and IFN‐ω levels in PLWH, regarding time‐points, there was no evidence of change in IFN‐α2 at T2 (*β* = −0.474, *p* = 0.230) or T3 (*β* = −0.317, *p* = 0.213), nor in IFN‐ω at T2 (*β* = −0.581, *p* = 0.096) or T3 (*β* = −0.289, *p* = 0.253). However, IFN‐β was significantly lower at T3 than at T1 (*β* = −0.541, 95% CI: −1.036 to −0.045; *p* = 0.032), though not at T2 compared to T1 (*β* = −0.460; *p* = 0.335, Table 4).

**Table 4:** Multivariable analysis of the factors influencing IFN‐I gene expression.

	INF‐α2			IFN‐β			IFN‐ω		
Variable	*β*	95% CI	*p*	*β*	95% CI	*p*	*β*	95% CI	*p*
T2 vs. T1	−0.474	−1.248 to 0.300	0.230	−0.460	−1.395 to 0.475	0.335	−0.581	−1.265 to 0.103	0.096
T3 vs. T1	−0.317	−0.815 to 0.182	0.213	−0.541	−1.036 to −0.045	0.032	−0.289	−0.785 to 0.207	0.253
Age (years)	0.004	−0.033 to 0.042	0.819	0.013	−0.020 to 0.046	0.429	−0.000	−0.036 to 0.036	0.999
Gender	−0.390	−1.051 to 0.270	0.247	−0.420	−1.000 to 0.160	0.156	−0.149	−0.784 to 0.486	0.646
CD4^+^ T cell count at T0 (cells/µL)	0.001	0.000 to 0.002	0.019	0.001	0.000 to 0.002	0.015	0.001	0.000 to 0.003	0.007
Nadir CD4^+^ T cell count (cells/µL)	−0.001	−0.003 to 0.001	0.325	−0.002	−0.003 to −0.000	0.033	−0.001	−0.003 to 0.001	0.292
Exposure to cART (years)	−0.008	−0.045 to 0.028	0.659	−0.013	−0.049 to 0.022	0.465	−0.001	−0.033 to 0.032	0.956
Baseline IFN at T0 (ln; same IFN as outcome)	0.124	−0.133 to 0.381	0.343	0.133	−0.074 to 0.341	0.208	0.115	−0.027 to 0.258	0.113
Days between measurements	−0.012	−0.019 to −0.005	0.001	−0.010	−0.017 to −0.003	0.005	−0.007	−0.013 to −0.001	0.023

Abbreviations: CI, confidence interval; Ln, natural logarithm.

CD4^+^ T cell count at T0 showed a statistically significant positive association with all three outcomes: IFN‐α2 (*β* = 0.001, 95% CI: 0.000–0.002; *p* = 0.019), IFN‐β (*β* = 0.001, 95% CI: 0.000–0.002; *p* = 0.015), and IFN‐ω (*β* = 0.001, 95% CI: 0.000–0.003; *p* = 0.007). On the contrary, nadir CD4^+^ T cell count was inversely associated with IFN‐β (*β* = −0.002, 95% CI: −0.003 to −0.000; *p* = 0.033) but not with IFN‐α2 or IFN‐ω (Table 4). Furthermore, time since measurement (days between assessments) was consistently associated with lower IFN levels: IFN‐α2 (*β* = −0.012 per day, 95% CI: −0.019 to −0.005; *p* = 0.001), IFN‐β (*β* = −0.010 per day, 95% CI: −0.017 to −0.003; *p* = 0.005), and IFN‐ω (*β* = −0.007, 95% CI: −0.013 to −0.001; *p* = 0.023).

Age, gender, exposure to cART, and the baseline (T0) IFN‐I levels were not significantly associated with changes in IFN‐α2, IFN‐β, and IFN‐ω over time (*p* > 0.05) (Table 4).

Additionally, the history of SARS‐CoV‐2 infection after the second dose does not affect the expression of IFN‐α2, IFN‐β, and IFN‐ω (IFN‐α2: *β* = 0.092, *p* = 0.761; IFN‐β: *β* = 0.285; *p* = 0.272; IFN‐ω: *β* = 0.296; *p* = 0.286) (Supporting Information S2: Tables 3 and 5).

## 4 Discussion

Given the impairment of adaptive immunity and the immune activation status in chronic HIV infection [14], assessing the immunogenicity of COVID‐19 vaccines in PLWH and their impact on innate immune mediators, including IFNs [26], warrants investigation.

Therefore, to increase the knowledge on the effects of mRNA‐based SARS‐CoV‐2 vaccine among PLWH, this study investigated its immunogenicity in PLWH who had received two or three doses of the Pfizer mRNA BNT162b2 COVID‐19 vaccine. Quantitative assessment of anti‐S IgG antibodies showed a progressive increase in anti‐S antibodies after each vaccine dose, comparable to those of healthy donors, which highlights the robust antibody production in PLWH following vaccination. The in vitro biological assay, which was based on the Vero E6/S‐VSV system, confirmed the longitudinal increase in anti‐S nAbs in PLWH. A small number of participants (5/75, 6.66%) showed detectable anti‐S IgG at baseline despite no reported history of SARS‐CoV‐2 infection prior to vaccination. However, as shown by the multivariable model reported in Table 3, baseline anti‐S IgG levels did not affect the longitudinal development of antibody responses. Therefore, these five PLWH were retained in the subsequent analyses.

Furthermore, the multivariable analysis revealed that the anti‐S IgG titers negatively correlated with the time interval between each measurement, indicating a physiological decline in antibody levels over time. Interestingly, the production of anti‐S antibodies was not influenced by age, gender, the duration of cART, nadir CD4^+^ T cell count, or CD4^+^ T cell count at T0. Moreover, neither a CD4^+^ T cell count of approximately 400 cells/µL nor the nadir CD4^+^ T cell count impacted the development of anti‐S IgG. This result is in line with previous reports [27, 28, 29], indicating that even PLWH with CD4^+^ T cell count below 200 cells/µL were able to develop RBD‐specific IgG following BNT162b2 COVID‐19 vaccination [27].

Previous studies have shown that IFNs modulate the magnitude and quality of antigen‐specific responses to mRNA vaccines. While IFN‐I does not impair particle‐mediated mRNA vaccination [30, 31], IFN signaling is crucial for antigen‐specific cellular immunity [32]; however, its role in BNT162b2‐induced adaptive immunity in PLWH remains unclear. Our findings suggest that the magnitude of anti‐S antibody production in PLWH is not affected by the expression of IFN‐α2, IFN‐β, and IFN‐ω genes prior to BNT162b2 vaccination. This is consistent with evidence that IFN‐I deficiency does not impair the B cell response to SARS‐CoV‐2 mRNA vaccines [33] and indicates that the status of innate immune activation is not a key determinant of humoral vaccine responsiveness in PLWH. Furthermore, the absence of significant correlations between IFN‐α2, IFN‐β, and IFN‐ω gene expression levels and both anti‐S IgG and anti‐S nAb levels at the corresponding time points does not support a direct association between IFN‐I responses and the magnitude of the vaccine‐induced humoral response over time in PLWH.

There is a significant lack of evidence regarding the mechanistic processes by which SARS‐CoV‐2 mRNA vaccination may influence the early activation of the innate immune response. All PLWH included in this study received the BNT162b2 vaccine, a nucleoside‐modified mRNA (modRNA) encoding the S protein as well as lipid nanoparticles containing ionizable lipids (iLNPs). These iLNPs ensure efficient cytoplasmic mRNA delivery and translation [34] and, beyond inducing protective nAbs [35, 36], can stimulate chemokines and pro‐inflammatory cytokines [37], reflecting their capacity to activate innate immune responses, including IFN‐I [34]. Similarly, other vaccines, such as those directed against influenza A virus, have been shown to induce a change in the IFN response, suggesting a link between IFN response and antibody response [38, 39, 40]. Although the modulation of the innate immune response to mRNA SARS‐CoV‐2 vaccination has been investigated in healthy individuals, data on vulnerable populations including patients with systemic lupus erythematosus [41], multiple sclerosis [12], and cancer [42], remain limited. In our study, a longitudinal analysis of IFN‐I in PLWH showed time‐dependent variations across the analyzed time point. While no changes were observed after the first BNT162b2 dose, IFN‐I transcript levels showed variation over the study period. Specifically, expression of IFN‐I genes was lower at T2 than at earlier time points, whereas IFN‐α2 and IFN‐ω, but not IFN‐β, were higher at T3 than at T2, indicating a time‐dependent variation in IFN‐I transcript levels. Furthermore, since most published studies on innate immune responses to SARS‐CoV‐2 vaccination have been performed in healthy individuals [43, 44], comparison with our findings should be made cautiously. In PLWH, persistent dysregulation of IFN‐related pathways may contribute to a different transcriptional background and may partly explain the limited longitudinal changes observed after vaccination. In addition, consistent with our previous work [45], comparison of the baseline IFN response in PLWH at T0 with that of healthy donors showed generally higher IFN‐I gene expression in PLWH, supporting the presence of HIV‐associated IFN dysregulation.

Nevertheless, the multivariable model, which was adjusted for demographic data, time of exposure to cART, immunological parameters, and the number of days between each time point, demonstrated that the IFN response remained unchanged across time points, except for IFN‐β, which was reduced at T3 compared to T1, suggesting possible long‐term, vaccine‐associated modulation of innate immune responses. Furthermore, a positive correlation was found between CD4^+^ T cell count and mRNA levels of IFN‐α2, IFN‐β, and IFN‐ω. This finding is consistent with previous evidence showing an association between immune status and IFN‐related transcriptional activity in cART‐treated PLWH [46, 47], while the relationship between CD4^+^ T cell count and IFN‐I transcript patterns remains poorly characterized in the specific setting of SARS‐CoV‐2 vaccination. Notably, PLWH who had experienced low nadir CD4^+^ T cell count, a known risk factor for AIDS‐ and non‐AIDS‐related complications and suboptimal immune reconstitution under cART [48], also exhibited a stronger IFN‐β response, potentially reflecting a compensatory enhancement of innate immunity.

Our study has several limitations. Although comparison with healthy donors at baseline showed higher IFN‐I gene expression in PLWH, consistent with HIV‐associated IFN dysregulation [45, 49], the lack of longitudinal IFN‐I gene expression data in the healthy donor group limits a more thorough and accurate interpretation of the temporal IFN‐I transcript patterns observed in PLWH. An additional limitation is the heterogeneity in post‐vaccination sampling times, particularly at T2, together with the absence of multiple closely timed post‐dose collections. Since IFN‐I gene expression is likely to be highly dynamic, this sampling scheme does not allow to define the kinetics of the IFN response and to distinguish BNT162b2 vaccine‐related effects from time‐dependent variability. A further limitation of this study is that IFN‐I gene expression was assessed in total PBMCs rather than in sorted cell subsets. Therefore, the observed transcript levels of IFN‐α2, IFN‐β, and IFN‐ω cannot be attributed to specific immune cell populations, and temporal changes may reflect both differences in gene expression within individual subsets and variations in PBMCs composition over time. Moreover, our analysis focused exclusively on IFN transcript levels and did not examine other inflammatory pathways following vaccination. Another limitation is that anti‐S nAbs were assessed only against the Wuhan strain without evaluating responses to other circulating variants. The study focused on PLWH receiving the BNT162b2 vaccine, thereby precluding the evaluation of the anti‐S antibody response and innate immunity elicited by other SARS‐CoV‐2 vaccine platforms.

In conclusion, BNT162b2 elicits anti‐S antibody responses in PLWH that are comparable to those in healthy donors, regardless of baseline or longitudinal IFN gene expression. Furthermore, our findings demonstrate that the expression of the IFN‐I genes remained unchanged following BNT162b2 vaccination in PLWH, with IFN‐β generally declining over time. Additionally, individuals with higher CD4^+^ T cell count demonstrate stronger IFN production, emphasizing the role of immune recovery in shaping the magnitude of IFN‐mediated immunity.

## Author Contributions

**Federica Frasca:** data curation, writing – original draft, investigation and methodology. **Luca Maddaloni:** data curation, writing – original draft, investigation. **Alessandra D'Auria:** investigation and methodology. **Matteo Fracella:** investigation and methodology. **Ginevra Bugani:** methodology. **Carmen Falvino:** methodology. **Valentina Baccolini:** formal analysis, writing – original draft. **Giuseppe Migliara:** formal analysis, writing – original draft. **Francesca Aloisi:** methodology. **Letizia Santinelli:** investigation. **Enrico Palermo:** investigation. **Luca Bortolani:** investigation. **Alessandro Lazzaro:** investigation. **Giancarlo Ceccarelli:** writing – review and editing. **Ombretta Turriziani:** writing – review and editing. **Claudio Maria Mastroianni:** writing – review and editing. **Guido Antonelli:** writing – review and editing. **Gabriella d'Ettorre:** writing – review and editing. **Carolina Scagnolari:** funding acquisition, data curation, writing – review and editing.

## Ethics Statement

This study involved human participants. The study protocol was approved by the institutional Review Board (Department of Public Health and Infectious Diseases, Sapienza University of Rome) and the Ethics Commitee (Comitato Etico Territoriale Lazio Area 1) (Ref. 6260, Prot. 0099/2021).

## Consent

Written informed consent was obtained from each study participant.

## Conflicts of Interest

The authors declare no conflicts of interest.

## Supporting information


Supporting File 1

Supporting File 2

## Data Availability

The data that support the findings of this study are available from the corresponding author upon reasonable request.
