# Supplementary material for: SARS‐CoV‐2 mRNA Vaccination Induces Neutralizing Antibodies and Type I IFN Changes in People Living With HIV
Source: J Med Virol. 2026 Jul 27;98(8):e71067. doi: 10.1002/jmv.71067 (PMC13402915; doi:10.1002/jmv.71067)
Supplement: Supplementary file 1 — Supporting File 1 [file JMV-98-e71067-s001.pptx]

## Slide 1
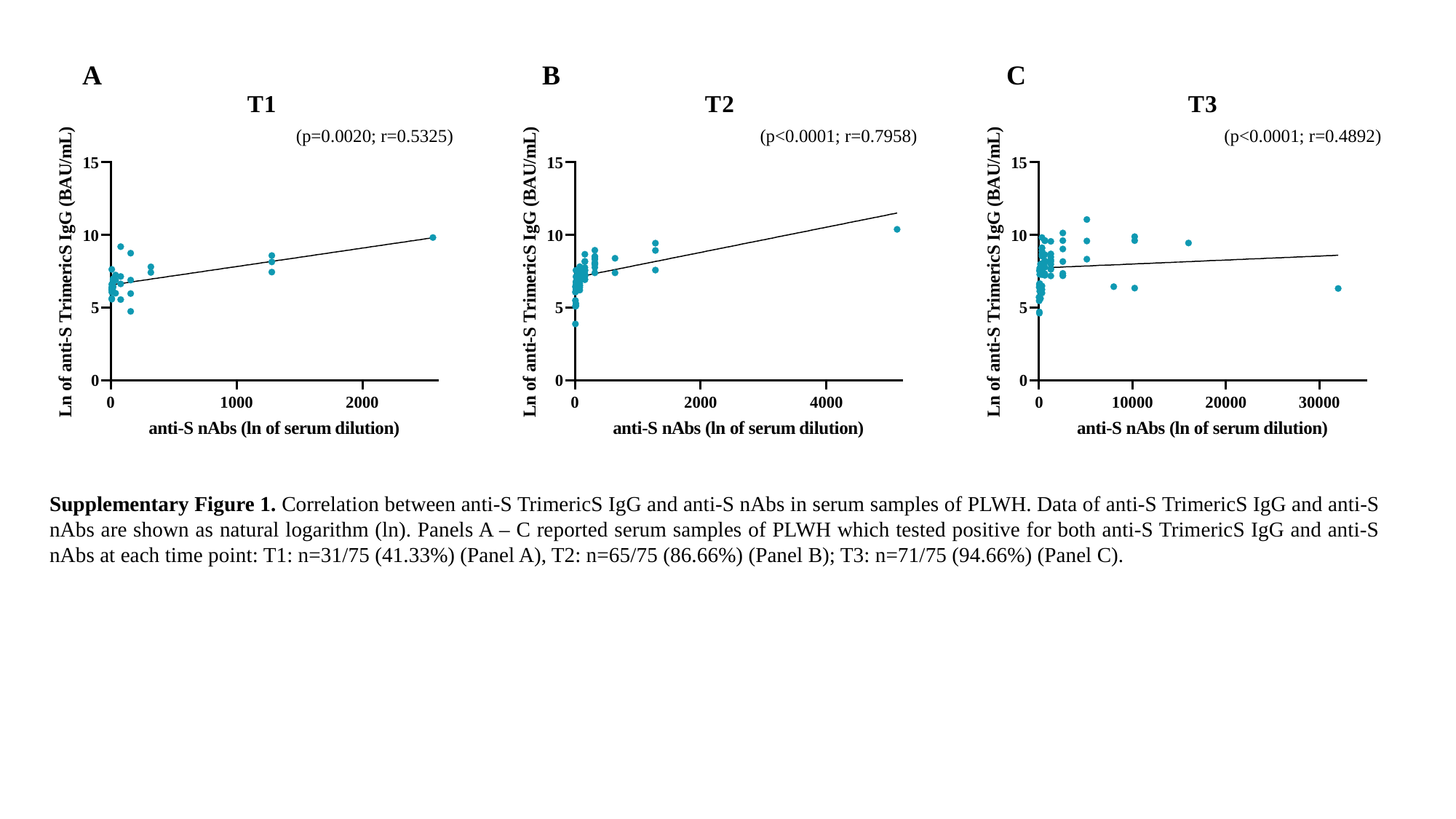

A
B
C
Supplementary Figure 1. Correlation between anti-S TrimericS IgG and anti-S nAbs in serum samples of PLWH. Data of anti-S TrimericS IgG and anti-S nAbs are shown as natural logarithm (ln). Panels A – C reported serum samples of PLWH which tested positive for both anti-S TrimericS IgG and anti-S nAbs at each time point: T1: n=31/75 (41.33%) (Panel A), T2: n=65/75 (86.66%) (Panel B); T3: n=71/75 (94.66%) (Panel C).

## Slide 2
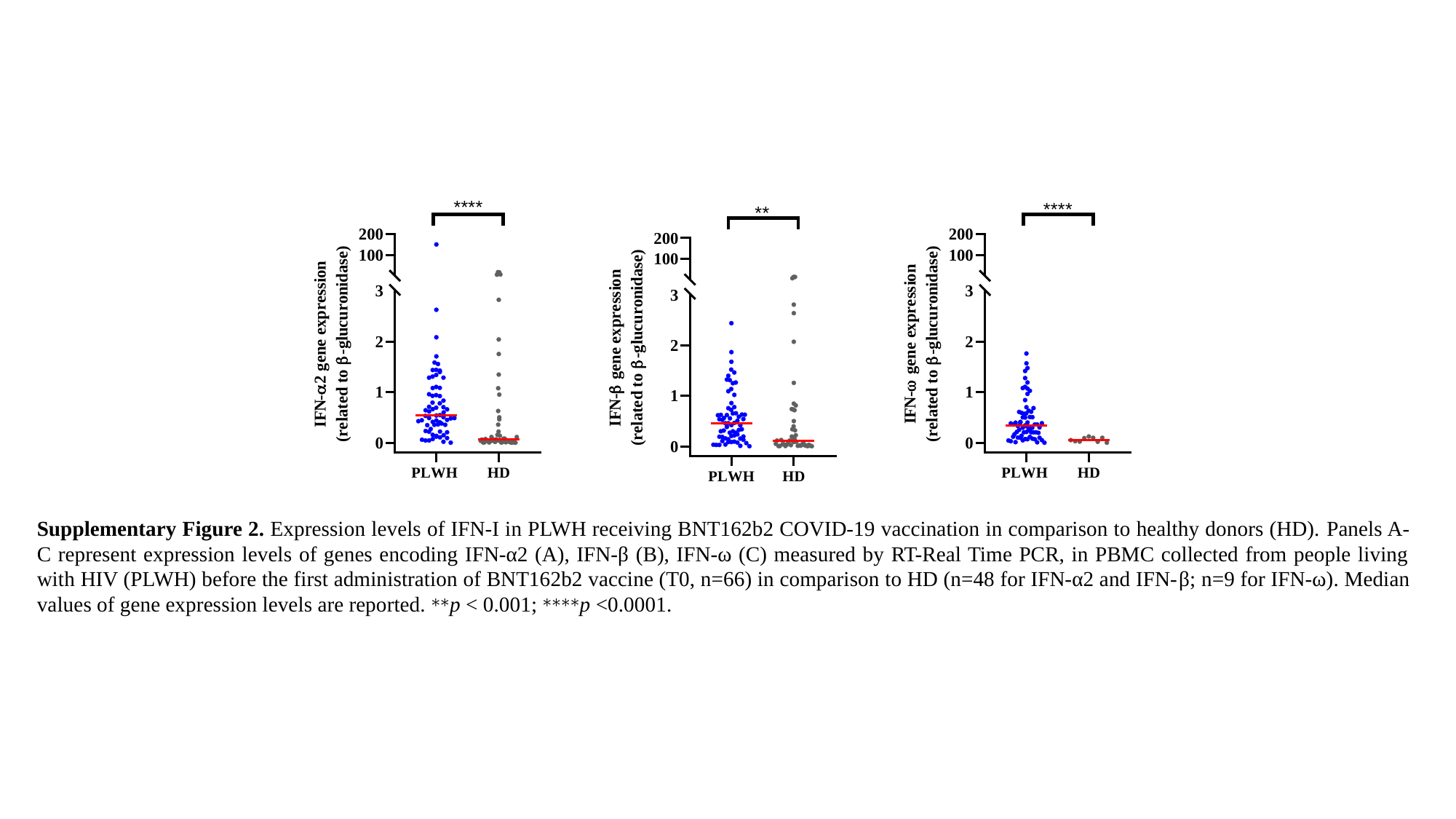

Supplementary Figure 2. Expression levels of IFN-I in PLWH receiving BNT162b2 COVID-19 vaccination in comparison to healthy donors (HD). Panels A-C represent expression levels of genes encoding IFN-α2 (A), IFN-β (B), IFN-ω (C) measured by RT-Real Time PCR, in PBMC collected from people living with HIV (PLWH) before the first administration of BNT162b2 vaccine (T0, n=66) in comparison to HD (n=48 for IFN-α2 and IFN-β; n=9 for IFN-ω). Median values of gene expression levels are reported. ∗∗p < 0.001; ∗∗∗∗p <0.0001.
