# Supplementary material for: SARS‐CoV‐2 mRNA Vaccination Induces Neutralizing Antibodies and Type I IFN Changes in People Living With HIV
Source: J Med Virol. 2026 Jul 27;98(8):e71067. doi: 10.1002/jmv.71067 (PMC13402915; doi:10.1002/jmv.71067)
Supplement: Supplementary file 2 — Supporting File 2 [file JMV-98-e71067-s002.docx]

| **Supplementary Table 1.** Multivariable analysis of the factors influencing anti-S IgG in PLWH | | | |
| --- | --- | --- | --- |
|  | *β coefficient* | 95% CI | *p-value* |
| T2 vs T1 | 1.714 | 0.933 to 2.495 | >0.0001 |
| T3 vs T1 | 1.839 | 1.349 to 2.330 | >0.0001 |
| IFN-α at T0 (ln-transformed) | 0.011 | -0.419 to 0.441 | 0.959 |
| IFN-β at T0 (ln-transformed) | -0.138 | -0.599 to 0.323 | 0.557 |
| IFN-ω at T0 (ln-transformed) | 0.119 | -0.047 to 0.286 | 0.160 |
| Age (years) | -0.012 | -0.040 to 0.015 | 0.381 |
| Gender | 0.294 | -0.211 to 0.800 | 0.254 |
| Nadir CD4+ T cell count (cells/µL) | -0.0004 | -0.002 to 0.001 | 0.645 |
| CD4+ T cell count at T0 (cells/µL) | -0.0002 | -0.001 to 0.0006 | 0.634 |
| Exposure to cART (years) | -0.007 | -0.037 to 0.022 | 0.620 |
| Days between measurements | -0.009 | -0.015 to -0.003 | 0.003 |
| Anti-S IgG T0 | 0.032 | -0.204 to 0.269 | 0.788 |
| SARS-CoV-2 infection after the II dose | 0.592 | 0.091 to 1.093 | 0.021 |
| CI: confidence interval. Ln: natural logarithm | | | |

| **Supplementary Table 2.** Multivariable analysis of the factors influencing anti-S IgG in PLWH | | | |
| --- | --- | --- | --- |
|  | *β coefficient* | 95% CI | *p-value* |
| T2 vs T1 | 1.338 | -0.106 to 2.782 | 0.069 |
| T3 vs T1 | 2.423 | 1.424 to 3.423 | >0.0001 |
| SARS-CoV-2 infection after the II dose | 0.691 | -0.299 to 1.682 | 0.171 |
| T2*SARS-CoV-2 infection after the II dose^#^ | 0.5041 | -0.817 to 1.826 | 0.455 |
| T3*SARS-CoV-2 infection after the II dose^#^ | -0.803 | -1.943 to 0.337 | 0.168 |
| IFN-α at T0 (ln-transformed) | 0.011 | -0.419 to 0.442 | 0.959 |
| IFN-β at T0 (ln-transformed) | -0.138 | -0.600 to 0.323 | 0.556 |
| IFN-ω at T0 (ln-transformed) | 0.119 | -0.047 to 0.286 | 0.160 |
| Age (years) | -0.012 | -0.040 to 0.015 | 0.381 |
| Gender | 0.294 | -0.211 to 0.801 | 0.254 |
| Nadir CD4+ T cell count (cells/µL) | -0.0004 | -0.002 to 0.001 | 0.649 |
| CD4+ T cell count at T0 (cells/µL) | -0.0002 | -0.001 to 0.0006 | 0.635 |
| Exposure to cART (years) | -0.007 | -0.037 to 0.022 | 0.621 |
| Days between measurements | -0.009 | -0.014 to -0.003 | 0.002 |
| Anti-S IgG T0 | 0.032 | -0.204 to 0.269 | 0.791 |
| CI: confidence interval. Ln: natural logarithm. ^#^ interaction between time points and history of SARS-CoV-2 infection | | | |

| **Supplementary Table 3.** Multivariable analysis of the factors influencing IFN-α2 gene expression | | | | |
| --- | --- | --- | --- | --- |
|  | *β coefficient* | | 95% CI | *p-value* |
| T2 vs T1 | -0.466 | | -1.239 to 0.306 | 0.237 |
| T3 vs T1 | -0.315 | | -0.814 to 0.182 | 0.214 |
| Age (years) | 0.004 | | -0.031 to 0.041 | 0.798 |
| Gender | -0.380 | | -1.029 to 0.268 | 0.250 |
| CD4+ T cell count at T0 (cells/µL) | 0.001 | | 0.000 to 0.002 | 0.031 |
| Nadir CD4+ T cell count (cells/µL) | -0.001 | | -0.003 to 0.000 | 0.288 |
| Exposure to cART (years) | -0.008 | | -0.045 to 0.028 | 0.647 |
| IFN-α at T0 (ln-transformed) | 0.121 | | -0.136 to 0.379 | 0.357 |
| SARS-CoV-2 infection after the 2^nd^ dose | 0.092 | | -0.503 to 0.688 | 0.761 |
| Days between measurements | -0.011 | | -0.018 to -0.004 | 0.001 |
| Anti-S IgG T0 | 0.082 | | -0.036 to 0.200 | 0.173 |
| CI: confidence interval. Ln: natural logarithm | | | | |
| **Supplementary Table 4.** Multivariable analysis of the factors influencing IFN-β gene expression | | | | |
|  | | *β coefficient* | 95% CI | *p-value* |
| T2 vs T1 | | -0.444 | -1.378 to 0.489 | 0.351 |
| T3 vs T1 | | -0.537 | -1.032 to -0.042 | 0.033 |
| Age (years) | | 0.014 | -0.017 to 0.047 | 0.370 |
| Gender | | -0.421 | -0.997 to 0.153 | 0.151 |
| CD4+ T cell count at T0 (cells/µL) | | 0.001 | 0.000 to 0.001 | 0.020 |
| Nadir CD4+ T cell count (cells/µL) | | -0.002 | -0.003 to -0.000 | 0.019 |
| Exposure to cART (years) | | -0.015 | -0.050 to 0.019 | 0.396 |
| IFN-β at T0 (ln-transformed) | | 0.132 | -0.075 to 0.340 | 0.211 |
| SARS-CoV-2 infection after the 2^nd^ dose | | 0.285 | -0.224 to 0.795 | 0.272 |
| Days between measurements | | -0.010 | -0.017 to -0.003 | 0.005 |
| Anti-S IgG T0 | | 0.105 | -0.080 to 0.291 | 0.266 |
| CI: confidence interval. Ln: natural logarithm | | | | |

| **Supplementary Table 5.** Multivariable analysis of the factors influencing IFN-ω gene expression | | | |
| --- | --- | --- | --- |
|  | *β coefficient* | 95% CI | *p-value* |
| T2 vs T1 | -0.567 | -1.248 to 0.112 | 0.102 |
| T3 vs T1 | -0.287 | -0.781 to 0.206 | 0.254 |
| Age (years) | 0.0014 | -0.033 to 0.035 | 0.935 |
| Gender | -0.144 | -0.777 to 0.487 | 0.654 |
| CD4+ T cell count at T0 (cells/µL) | 0.001 | 0.000 to 0.002 | 0.007 |
| Nadir CD4+ T cell count (cells/µL) | -0.001 | -0.003 to 0.000 | 0.187 |
| Exposure to cART (years) | -0.002 | -0.035 to 0.030 | 0.872 |
| IFN-ω at T0 (ln-transformed) | 0.112 | -0.027 to 0.252 | 0.114 |
| SARS-CoV-2 infection after the 2^nd^ dose | 0.296 | -0.247 to 0.840 | 0.286 |
| Days between measurements | -0.007 | -0.013 to -0.001 | 0.020 |
| Anti-S IgG T0 | 0.140 | -0.022 to 0.303 | 0.092 |
| CI: confidence interval. Ln: natural logarithm |  |  |  |

| **Supplementary Table 6.** Correlation between anti-S IgG and IFN-I gene expression | | | |
| --- | --- | --- | --- |
| Time points | T1 | T2 | T3 |
| Item | Anti-S IgG | Anti-S IgG | Anti-S IgG |
| IFN-α | p=0.4124 | p=0.1414 | p=0.8403 |
|  | r=-0.1042 | r=0.2518 | r=0.02656 |
| IFN-β | p=0.5857 | p=0.0562 | p=0.8729 |
|  | r=-0.06942 | r=0.2363 | r=0.02109 |
| IFN-ω | p=0.3590 | p=0.1944 | p=0.8679 |
|  | r=-0.1166 | r=0.1618 | r=-0.02192 |
| Anti-S IgG: anti-Spike IgG | | | |

| **Supplementary Table 7.** Correlation between anti-S nAbs and IFN-I gene expression | | | |
| --- | --- | --- | --- |
| Time points | T1 | T2 | T3 |
| Item | Anti-S nAbs | Anti-S nAbs | Anti-S nAbs |
| IFN-α | p=0.5427 | p=0.2432 | p=0.4770 |
|  | r=-0.07750 | r=0.1457 | r=-0.09357 |
| IFN-β | p=0.5889 | p=0.1302 | p=0.3849 |
|  | r=-0.06882 | r=0.1882 | r=-0.1142 |
| IFN-ω | p=0.6333 | p=0.3730 | p=0.3193 |
|  | r=-0.06077 | r=0.1115 | r=-0.1308 |
| Anti-S nAbs: anti-Spike neutralizing antibodies. | | | |
